# Perceptions of death and memory transmission among residents of Hiroshima and Nagasaki, Japan: A qualitative study

**DOI:** 10.1371/journal.pgph.0002061

**Published:** 2023-08-31

**Authors:** Monte-Angel Richardson, Carly Parmer

**Affiliations:** 1 Faculty of Social Work, University of Toronto, Toronto, Canada; 2 English Department, Nagasaki Technical High School, Nagasaki, Japan; The Valens Clinic, UNITED ARAB EMIRATES

## Abstract

The understanding and materialization of grief and loss in a community are contingent upon cultural norms, historical processes, and dominant political narratives. The processes of public mourning create a localized memory of the deceased which contributes to a collective narrative formation around loss. When death is made public, politicized, or collectively grieved, there exists great momentum for enacting policy change through restorative justice practices. This momentum for resistance is amplified when collective grieving takes place following political or mass deaths. The present study aims to develop a holistic understanding of mourning and memorialization practices as they are locally enacted in Hiroshima and Nagasaki, Japan. These two cities were chosen based on their shared history of mass violence and their diverging cultural customs of mourning. Twelve qualitative interviews were conducted with residents of both cities. The purpose of the interviews was to gain insight to how Hiroshima and Nagasaki residents make meaning out of loss and public memorialization. Narrative interviews based on the Miyabayashi Grief Measurement (MG) allowed participants to explain and reflect on the links between their public and individual mourning practices. Themes identified in the interviews include (1) a routine and automatic engagement with grief rituals specific to Japanese culture; (2) connection and gratitude towards ancestors; (3) methods of engaging with memorial sites to transmit personal memories of the deceased; (4) a sense of duty in passing on the first-hand accounts of survivors of the atomic bombing; (5) recalling memories of the deceased when making decisions; and, (6) transmitting memories of loss in a way that is celebratory and joyous. These results ask us to look past simplified depictions of cultural grief and consider the individual elements that may impact a person’s remembrance and memory transmission within societies.

## Introduction

When a traumatic event occurs, it shakes the foundations of a society, and most people can remember where they were, or what they were doing, when that event took place [1, 2]. Research has revealed that individuals and communities come together following traumatic events. The impacted communities engage in memory work in response to the traumatic event such as public memorials, monuments, policy change, or the formation of groups to bring the community closer together [3–5]. As time progresses, these personal memories collide and combine to create widely known larger narratives which tell the stories of both the survivors and the lives lost to the traumatic event These narratives are consecrated by the establishment of public remembrance activities, stored within societal institutions ranging from governmental organizations to memorial sites. For example, research has been conducted regarding the use of war memorials to forward political agendas, including monuments and museums [6]. Such memorial sites consecrate and make known the intricate interweaving of cultural memory and identity.

The process of developing a shared memory around events has been termed collective memory [7, 8]. Collective memory entails the narration and representation of the past, where nations, collectives, and individuals turn to words and images to ensure that certain events, eras, people and experiences are remembered. Since its inception, the study of collective memory has focused on both the actions of societies and communities to maintain public symbols related to memory, such as memorials, and how individuals form and maintain collective memories through their actions and behaviors [6, 7, 9, 10]. Through the processes of collective remembering or forgetting, public regard can either strengthen or weaken the original memories held about the event. The methods by which individuals transmit and share memories of those who have died have important implications for the collective memories about the perspectives of death and mourning within a community. This is doubly true for places impacted by a legacy of mass violence and trauma. In such places, collective remembering can lead to restorative justice to individuals impacted by political violence, such as the efforts that took place following the Pinochet dictatorship in Chile and the Dirty War in Argentina [11]. In the case of these two countries, collective memory catalyzed restorative justice advocacy for the survivors of these violent political regimes, offering memorials to the victims and providing a window of opportunity for policy changes which ensured that such regimes could not take power once again [12].

To understand how collective memories are formed, scholarly attention is now being paid to the transmission of memories between individuals and groups [13, 14]. Coman et al have found that, in sharing their experiences of an event, groups of people come to create a shared narrative of an event [7]. This narrative is created through the process of conversation, wherein individuals compromise or assert their own descriptions of how the events transpired through a process termed socially shared retrieval-induced forgetting (SSRIF). These suggestions are accepted or rejected by the entire group, eventually forming a shared narrative of the events. Research of memory transmitted through conversation has been validated by Hirst & Coman [2, 7]. In their research, these psychologists have uncovered the different roles that individuals take in group dynamics which contribute to the transmission of memory. In a study of memory recall among Princeton students (n = 128), similar patterns of selective forgetting were found to be more likely to occur between speakers and listeners if they perceived as belonging to the same social university, indicating that group membership and identity were central to memory recall [2].

Studies pertaining to collective memories of grief have focused on various aspects of rituals related to sharing memories about the deceased. Castle et al define grief rituals as specific behaviors or activities which give symbolic expression to certain feelings and thoughts of actors individually or as a group [15]. Rituals in this sense may be performed once or on a continual basis. Morioka et al echo this definition by contrasting Japanese grief rituals with those of Western cultures, which make a clear distinction between death and life, and noting that in Japan, there seems to be a recognition of continuity between these two states. In Japan, there is a belief that a person’s spirit belongs to the same family and the same local community before and after the death. The authors propose that this belief about death may influence the feeling of attachment between the bereaved and the dead, helping people grieve and transmit these memories in a peaceful way [16].

Other studies discussed the ways that death was remembered, such as through rites of passage, where death is described as a transition between the two states of being alive and dead [17]. Klass went into detail about the processes of grieving established in Japan, discussing the history of ancestor rituals and their connection to national allegiance [18]. He dates the reverence for ancestors back to the Nara era in Japan, the development of which was based on a desire to link imperial ancestors to one’s own ancestors. Yamamoto et al define ancestor worship as processes of mourning based on a continuing presence of the dead [19].

A handful of studies have focused on the methods of transmitting memory among individuals who have experienced trauma, such as survivors of World War II, and specifically the atomic bombing of Hiroshima and Nagasaki in 1945. Yoshida et al focus on both Hiroshima and Nagasaki as case studies to explore the extent to which sites of atomic bombings have been memorialized and embedded in tourism promotion for educational purposes [20]. In their interviews with memorial site staff and volunteers, the authors found that the atomic bombing stories delivered by hibakusha were an important part of the itinerary for tourists to both cities. Regarding the memory transmission of these events to tourists, the authors note that the aging of hibakusha has challenged museum management in both cities to maintain a forum where hibakusha can share their survival experiences [20].

A case study was similarly conducted by Foard about the universal aspects of mourning the atomic bombing in Hiroshima [17]. In this case study, Foard juxtaposes the proceedings of mourning that are typical in Japan with those specific rites conducted in Hiroshima following the atomic bombing of 1945. One unique characteristic found regarding mourning the atomic bombing in Hiroshima was the making universal the processes of grieving that have typically been familial and local. As a result, private expressions of grief served as national and global metaphors regarding the impact of the bombing [17].

While these findings contribute to the body of knowledge of collective memory, not much scholarly attention has yet been paid to the transmission of grief memories undertaken by individuals across generations, particularly among those living in a place impacted by a legacy of mass violence. The purpose of this paper is to contribute to the body of literature regarding the methods of transmitting grief memories and mourning processes among individuals living in a place impacted by mass violence. Participants of the study are individuals from the cities of Hiroshima and Nagasaki in Japan, the first two places in the world to be impacted by atomic bombing. It is believed that the legacy of the bombing will be visible in the individual’s conceptualization of death and loss, in their mourning rituals, and in their association to the larger community.

## Key definitions

In order to communicate effectively the findings of this study, key terms prevalent in the thesis are here defined.

### Atomic bombing of Hiroshima and Nagasaki, 1945

Several terms related to the atomic bombing of Hiroshima and Nagasaki are prevalent in this thesis. One often used term is ‘hibakusha’, which is the Japanese word for survivors of the atomic bombing. Another related term is ‘second-generation bombing survivor’, which indicates that this is a person whose parents were survivors of the bombing. Another term, ‘close distance survivor of the bombing,’ indicates a person who was near or within the hypocenter area during the atomic bombing in either city.

### Obon

Frequently referenced in the paper is Obon (お盆) or Bon (盆), a Japanese Buddhist custom to honor the spirits of deceased ancestors. This custom has evolved into a family reunion holiday during July or August, depending on the region. During this holiday, Japanese people return to ancestral family places and visit and clean their ancestors’ graves, and it is believed that the spirits of the ancestor’s revisit household altars and gravesites during this time. It has been celebrated in Japan for more than 500 years and there are many region-specific traditions, such as Bon Odori, a traditional dance that takes place differently based on region.

## Methods

### Ethics statement

Several steps were taken to reflect on the research team’s positionalities in relation to the study. First, the PI completed three courses focused on the ethics of global engagement through social justice, diversity, and oppression practice in social work. These courses were completed as a requirement for a grant which funded the study. Second, the PI hired a fluent Japanese-speaking interpreter to assist during the interviews and a translator to assist in the development of interview transcripts. Third, all pertinent members of the research team completed personal reflections by the end of the study. In addition to these, the PI completed four personal reflections during the study process and echo interviews following each interview.

The study team consisted of members who identified in differing ways as either ‘outsiders’, ‘insiders’ or some combination of the two. The principal investigator, as neither a fluent Japanese speaker nor citizen, considered herself to be a ‘pure outsider.’ The community partner in Nagasaki, as a US citizen but Japanese speaker residing in Japan for several years, considered herself to be an ‘outsider-in.’ Both academic affiliates in Hiroshima and Nagasaki, as native Japanese speakers and citizens, considered themselves to be ‘insiders’ who were members of the communities. Lastly, the interpreter identified as being a white person who was born in Japan and spoke fluent Japanese, and considered herself to be an ‘insider-out’. These distinct positionalities were discussed at research team meetings. The research team believed that their differing positionalities allowed for detailed reflections throughout the study which added complexity and unique insights at every part of the process.

These positionalities allowed for unique engagement with participants and the data in three important ways. First, participants were able to speak freely in Japanese due to the presence of the interpreter. The PI’s conversational language abilities in Japanese and understanding of cultural nuances in communication also facilitated dialogue about the nature of the study and to answer questions. Second, the unique perspectives of members of the study team contributed both to the recruitment of participants and analysis of data. For example, the professors in both Hiroshima and Nagasaki provided feedback regarding interviewing a diverse age range of participants and facilitated engagement with these individuals. The study team also provided unique insights on the specific nuances of interviews during their personal reflections, and these were incorporated into the discussion of qualitative themes during data analysis. Third, a community partnership was made between the PI and faculty of social work in Hiroshima following the study, wherein the PI provided an evaluation of a community program being implemented at the university along with a presentation given to students of the social work master class. These exchange of skills and services allowed for a continued partnership which is still present to this day.

It is no simple task to engage with members of a different country and culture to discuss difficult things like grief and loss. However, with the guidance of experienced research faculty this study was able to be conducted with active engagement from the research team, who hold unique positionalities in relation to the cities of Hiroshima and Nagasaki. The additional data of personal reflections and echo interviews facilitate a reflexive process for all members of the research team engaged with the study at different phases of the process. This approach was useful not only for the recruitment and engagement with participants, but in the interpretation and analysis of the interview data.

### Participants and procedures

Following receipt of Institutional Review Board approval of this study at the University of Michigan, qualitative survey data were collected through narrative qualitative interviews (n = 12). Data were collected June and July of 2018. Participants were recruited after establishing relationships with faculty and staff at Nagasaki University and the Prefectural University of Hiroshima. Academic partners from both universities assisted with participant recruitment and the development of data collection methods. Participants were paid ¥2190 Yen, which was the equivalent of US $20 at the time of data collection. Interviews were conducted in person and lasted 30 minutes maximum. The interviews were conducted in Japanese with assistance from an interpreter. The interview questions were conducted in a semi-structured format, and audio recordings of the data were kept for analysis purposes.

Twelve interviews were conducted with adult participants from Hiroshima (n = 6) and Nagasaki (n = 6). Participants ranged in ages 18 to 90, and five participants identified being over the age of 80. Exactly half of respondents identified as male and half identified as female. All participants were residents of Japan and identified as ethnically Japanese. Of these participants, four identified as first-hand survivors of the atomic bombing, otherwise known as *hibakusha*. All names of participants are altered to protect anonymity.

### Miyabayashi Grief Measurement (MGM)

Interview questions were created based on the Miyabayashi Grief Measurement (MGM), a quantitative survey tool created to measure levels of grief specifically for Japanese people [21, 22]. The MGM is a 26-item questionnaire consisting of four subscales: mood stability, adaptive effort, cherished reminiscence, and alienated feelings. To acquire information about the public aspect of mourning, an additional category of public memorialization was also included. Inclusion of public memorialization in the conceptualization of collective grief is well supported in sociology, social work, and psychology literature [5, 8, 15, 23]. Participants were asked an even number of questions from each dimension (see: Table 1: Interview Topic Guide).

**Table 1:** Interview topic guide.

Background 過去の経験	Cherished Reminiscence 過去の回想	Daily Life 日常生活	Familial 家族	Public 社会一般
Have you ever known someone that has died? これまでに身近な誰かがなくなっていますか?	What are some things you enjoy doing that remind you of those that have passed on? あなたが日常生活の中で、何か良い事ををきっかけに亡くなった大切な人のことを思い出すことはありますか?	How do you feel that mourning impacts your daily life? 喪に服すことが日常生活に影響を与えることはありますか?もしあるならどんな影響ですか?	Does your family have a family gravesite? 家族の墓 (地)はありますか? どのくらいの頻度でお墓参りに行きますか? お墓参りに行くとどのような気持ちになりますか?	How do you commemorate the lives lost from the atomic bombing? あなたはに原爆の日をどのようなこととして記憶にとどめていますか?
How much time has passed since the death of someone close to you? 身近な方が亡くなられてからどれくらい時間が経ちましたか?	Did you feel like your family and friends supported you through dealing with the loss of someone you know? If yes, how do feel they supported you most? あなたにとって親しい方が亡くなった時に、友人や家族が支えになったように感じましたか?もし感じたなら具体的にどのような事が最も支えに感じましたか?	Do you feel upset by memories of the person you lost? 亡くなった方とのことを思い出して、気もちが動揺することがありますか?	Does your family have a family altar at your home? あなたの家には仏壇あるいは家族の祭壇がありますか? どのくらいの頻度で仏壇を拝みますか? 仏壇を拝むとどのような気持ちになりますか? (祭壇を訪問しますか?どのような気持ちになりますか?)	Have you ever visited _____ (Suwa Shrine, Sakamoto International Cemetery, Nagasaki Peace Park)? ——を訪れたことはありますか? (平和記念公園、原爆ドーム、原爆資料館)
What did this person mean to you and to this community? あなたや家族、地域(コミュニティ)にとってその方はどのような存在でしたか?	When you think back about this person(s), what memories come to mind? あなたが亡くなった方のことを考える時、どんな記憶を思い出しますか?	If yes, what activities or rituals help you feel better about the loss? Or, what activities or rituals help you feel connected to the community? 答えが「はい」の場合、どのような活動や儀式が、コミュニティとのお繋がりを感じさせますか?	If yes, how often do you visit the gravesite/altar? ある場合は、墓地/祭壇をどのくらいの頻度で訪問しますか?	Why do you think it was important to visit _______ (Suwa Shrine, Sakamoto International cemetery, Nagasaki Peace Park)? なぜ——を訪れることは重要性だと考えますか? (平和記念公園、原爆ドーム、原爆資料館)

### Analytical approach

This study employs thematic coding procedures in analyzing text chunks from narrative interviews to understand how participants transmit memories about death and loss, along with their personal practices of expressing grief. Narrative interviews allowed participants to explain and reflect on the links between their public and individual mourning practices. Thematic coding was selected due to it being previously used to analyze individual narratives and memories [2]. Additionally, thematic coding has been well supported in social work literature and research as a method to understand individuals’ meaning-making processes. For the purpose of this study, data collected in Hiroshima and Nagasaki are juxtaposed and compared in analysis.

**Fig 1:**
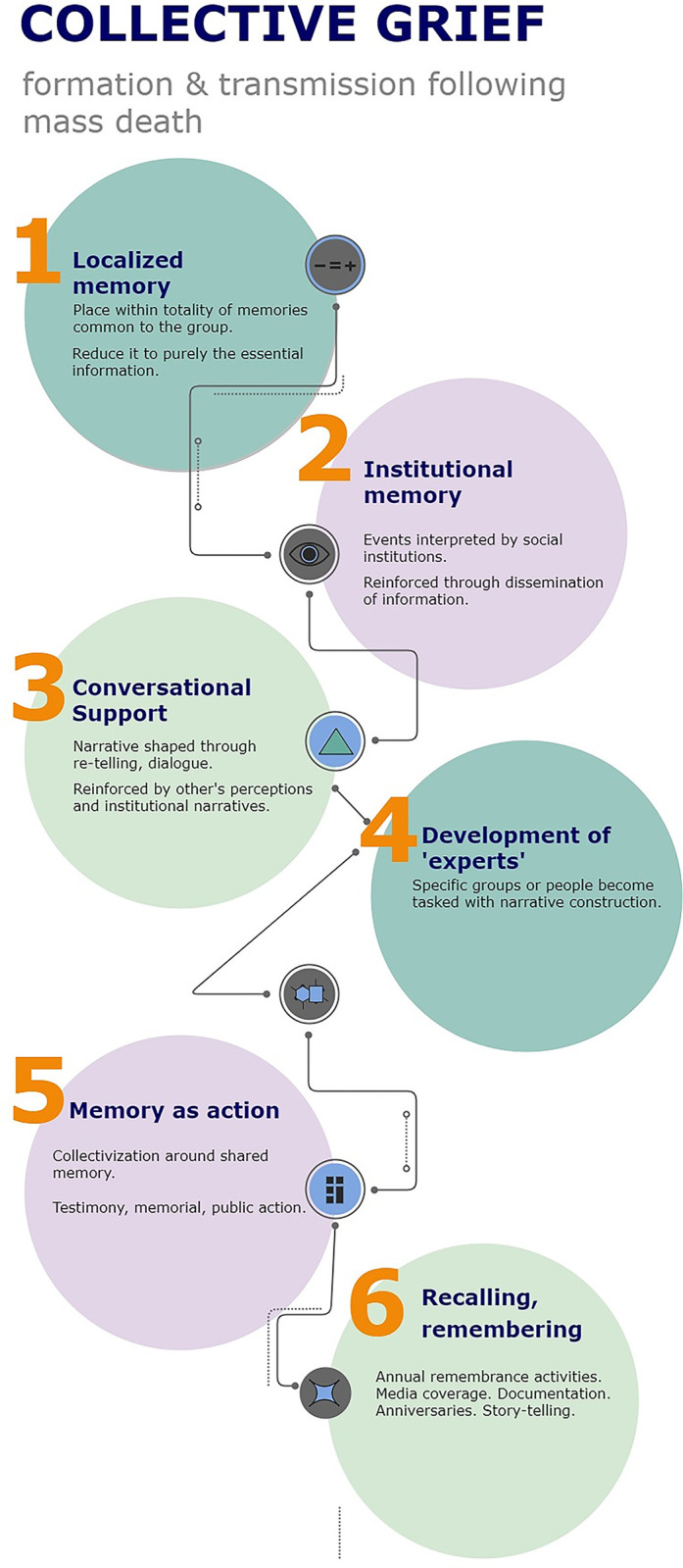
Collective grief framework: Formation and transmission following mass death.

Questions from the public dimension pertained to the participant’s commemorative mourning activities. These include their commemoration of the lives lost to the atomic bombing, their experiences visiting family gravesites, and plans for Obon. Questions from the familial dimension pertained to the participant’s feelings about support received from family during their grief processes. Questions were also asked about whether the participant had recently lost a close family member or friend. Questions from the personal dimension asked participants of their individual mourning practices and feelings about loss. Questions were asked on personal, familial, and public dimensions in order to understand how the individual weaves these three dimensions together when transmitting memories of loss and death. A flow chart was developed to visualize the flow of transmission of the collective grief experiences from the different domains (see: Fig 1: Collective Grief: Formation and Transmission Following Mass Death.).

### Positionalities of research team

Given the highly sensitive and emotional nature of the interviews conducted, it was imperative that the research team engage ethically and equitably with participants while reflecting on their own positionalities. The principal investigator is not a resident of Japan and speaks Japanese conversationally, but not fluently. Her interest in the research topic stems from her own experiences of mass trauma following a mass shooting at her alma mater, UC Santa Barbara. Following the traumatic event, the PI noticed that the processes around grieving and memorializing the dead changed for herself and many others within the impacted community. The PI wanted to ascertain if these experiences were felt among other survivors of mass trauma, across generations and in different localities.

Japan was selected as the focus of the current study due to the PI’s connections with community leaders and researchers in both Hiroshima and Nagasaki, two communities impacted by a legacy of mass violence. One faculty member at Hiroshima Prefectural University is an affiliate of the PI’s alma mater and ethical oversight institution, the University of Michigan. When considering conducting the study, the PI met with this faculty member and discussed possible research methods, approaches, and cultural nuances relevant to working with individuals in Hiroshima, Japan. One of the PI’s colleagues was also working in Nagasaki as a teacher for four years when the study was initiated, and this colleague became a community partner who assisted with participant recruitment, data collection, and data analysis in this city. The community partner introduced the PI to a faculty member at Nagasaki university who assisted with study design and data collection in this city.

## Results

Themes identified in the interviews include (1) routinized engagement with grief rituals specific to Japanese culture; (2) conveying a sense of connection and gratitude towards ancestors; (3) methods of engaging with memorial sites to transmit personal memories of the deceased; (4) a sense of duty in passing on the first-hand accounts of survivors of the atomic bombing; (5) recalling memories of the deceased when making decisions; and, (6) transmitting memories of loss in a way that is celebratory and joyous.

### Routinized engagement in grief rituals

The sentiment that mourning practices were routine, ritualized, or a natural thing to do was shared by a majority of participants from both Hiroshima and Nagasaki (n = 10) and was found among participants of differing ages and genders.

Kumiko, a survivor of the bombing from Hiroshima, first described feeling unsure of the reasons why she felt that Obon was important, noting that she had never previously thought of this. Upon reflecting further, she identified that the connectedness she felt with her ancestors was able to be expressed and shared with other family members during Obon. Kumiko expressed surprise when asked about her reasons for attending Obon, followed by a reflection that she had not previously considered the reasons behind participation. This recognition of her own complexity enabled her to articulate the feelings of connection and gratitude that motivated her prayer and participation in Obon rituals.

I: So, do you feel that participating in Obon is important for mourning and grief? So does it help with you grieving and experiencing loss?

    K: Very important!

    I: Why might that be?

    K: Why!?

K: I’ve always thought it important but I never really thought about the reason. I guess it’s more of a routine now.

For Marika, a second-generation survivor of the bombing in her mid-70s from Nagasaki, connecting with ancestors meant engaging in a daily prayer. She did not necessarily feel that prayer was an important part of her daily life as much as it was a part of her routine.

M: It’s not something that’s very important in my daily life. It’s really part of my routine—I don’t know if that’s the right way to say it. It’s like we have a custom to pray in the morning. We’re not constantly praying.

While many participants noted that they often thought of those who passed, the practice of mourning itself was not viewed as a spontaneous activity. A component of these routines was engaging in mourning activities at specific and designated times and dates.

Motoki, a 35-year-old male resident from Nagasaki, for example, described how he rarely reflects on those who have passed outside of the specified dates when ancestor rites were customarily performed for Buddhist rituals.

M: I don’t think they [mourning processes] often have influence. But for example, in Japan when you die there’s a funeral, and a week later there “Shonanoka” (7th day service). And then after “Shijuukunichi” (49th day service), next is “Sankaiki” (third anniversary). I think that there’s all of these timings to grieve. There are only events on these days where we get together and talk about the past, that’s about it.

Earlier in the interview, Motoki listed seven people he has personally known who have passed away, including family members and friends. Despite knowing many people who have passed, Motoki did not feel that mourning processes played a significant role in his life. Instead, he regarded the customary times for mourning as appropriate for expressing his grief.

The practice of grieving at specific and designated times was extended to grieving the lives lost to the atomic bombing of 1945. Participants reflected that they planned to attend specific events planned for commemoration, including a moment of silence broadcasted on television and annual events taking place at memorial parks on the day of the bombing. Yumi, a survivor of the atomic bombing from Hiroshima, described an experience of mourning the death of her husband at specific anniversaries and dates. These processes were viewed by Yumi to be important to be conducted in a specific and regularized fashion.

Y: Though my husband has no relation to the bombing. He came back and we got married. As of this year it’s been 18 years since he passed. For 15 years I visited his grave on the 13th of every month, the day of his death.

    I: And that was every month?

Y: Every month. I also made sure to do the proper ceremonies during New Year’s and the 7th, 23rd, and 27th anniversaries of his death. Other than that, I made sure there were fresh flowers before the anniversary of his death, on the 12th or 13th or sometimes 11th. I was usually punctual. And someone from the temple came to our house until the 7th anniversary.

Yumi also spoke of wishing she had taken better care of her husband while he was still alive, as he was often sick.

Y: Well, now I don’t feel so strongly but for a long time, I felt like I should have looked after him better, even though I was trying to do my best at the time, things kept getting in the way. And I say thank you for keeping me healthy through his protection, always. Well I say that every day, in the evening. Every evening I quickly say thank you, I was able to get through the day in good health because you protected me.

This concern for her deceased husband resulted in Yumi’s transmission of her memory through engagement in daily prayer typical mourning rituals following his death. Hence, Yumi’s experiences of grief were communicated through the established and available customs of prayer and visiting her husband’s gravesite. Additionally, the existence of such rituals in Japanese society allowed her to communicate her grief in a way that was easily accessible. As Hiro, a 19- year-old undergraduate student from Hiroshima, stated,

H: When someone has passed away, maybe the Obon after that we may talk about their memories, but after 5, 10 years we don’t hear things like that anymore. I myself have never experienced the death of someone I know so I can’t really say, but that’s what I hear.

Without having ever experienced death personally, Hiro was able to articulate the anniversaries and specific dates that were designated for the transmission of memories of the deceased between family and individuals. Participants easily described the intentionality of these mourning practices and the comfort they felt when engaging with them.

### Connecting to ancestors

Participants residing in both cities reflected that their engagement with grief practices, including prayer and visiting memorial sites, resulted in feelings of connectedness with deceased ancestors. They reflected a belief and hope that ancestors would protect living members of their family. These sentiments were particularly emphasized when engaging in events where this was expected, such as praying at a family altar or during Obon.

Additionally, participants described feeling gratitude towards ancestors, viewing their lives as possible because of their lineage which connects them. Kumiko, an 80-year-old survivor of the atomic bombing from Hiroshima prefecture, for example, reflected on gratitude towards her ancestors and described how this feeling motivated her to transmit this feeling through prayer.

K: Come to think of it, this life was given to me through generations and generations of ancestors, so I feel a connection and gratitude that I want to convey through prayer.

### Engaging with memorial sites

A major component of memory transmission discussed by participants was experiences had at memorial sites. Participants reflected on the role that experiences of learning played in shaping their connections between memorial sites and grieving. Residents of both cities were involved in educational activities that were instrumental in their articulation of the importance of certain sites. This was particularly true regarding sites dedicated to the atomic bombing of 1945, where participants often found opportunities to increase their personal awareness and knowledge of the tragedy. Visiting these memorial sites also encouraged participants to recall and transmit memories of their personal losses.

Akihiro, for example, used his nuanced knowledge of World War II to challenge narratives that excluded ‘others’ and forgotten victims of the atomic bombing and the war.

A: As for the Peace Park, there are several reasons why I go there. Of course, it was the center of the atomic bombing and the park north of the hypocenter itself is important to visit. There are many monuments in the park though, and even just going around to look at all the monuments takes time, but I understand that although it is true that there were many Japanese victims who suffered and whose lives were lost in the war, Japan was very active in the WW2, and they even tried to occupy China and Korea. Because of that, many Korean and Chinese people were brought to Nagasaki against their will and suffered from the bombing as well and there is a monument dedicated to those people in the park. Those Korean and Chinese people mustn’t be forgotten. It’s not just the Japanese who suffered.

Because Akihiro was familiar with memorial sites dedicated to these groups, he felt it was important to highlight the exclusion of Chinese and Korean victims of the war when remembering these events.

Hiro, a 19-year-old undergraduate student from Hiroshima, similarly used his educational experiences as a tool for transmitting a specific perspective about the purpose of Hiroshima Peace Park.

H: I myself have never experienced the atomic bombing, but ever since elementary school we have had school on that day despite it being summer vacation. This is currently being debated as a problem actually, and we’ve learnt about the atomic bombing and the importance of peace. Even though I didn’t experience it first hand, even as an elementary school child, I could tell that it was a very special day, and I knew everyone else felt the same way I did. On the day of the bombing there are several broadcasts on TV showing the peace memorial ceremony and I think people all over the world know this day as the day that a very important event took place.

For Hiro, sharing his perspective of the Peace Park meant conveying the experience of feeling that others shared similar views about its importance. He noted that his elementary school education was important for developing his awareness of the significance of the Peace Park and the day of the bombing. Hence, the education system itself became central to predicting how Hiro would transmit the memory of these events as an adult. Highlighting the importance of the day of the bombing by requiring that students attend school on that day was important for instilling a sense of importance for Hiro and his classmates.

Marika, an 80-year-old second generation survivor of the atomic bombing from Nagasaki, expressed the belief that different places in Nagasaki Peace Park held different meanings for her. By praying at the hypocenter where victims died during the bombing, Marika identified a personalized method of remembering that was significant for her transmission of grief. She emphasized that praying at the hypocenter felt more authentic to her mourning processes, indicating a localized relationship with the memorial sites that differed from the mainstream. Hence, the societal mechanism of the memorial site influenced Marika’s grieving and processes of transmitting memory about loss.

Many participants reflected on the ways their personal or professional roles impacted their forms of engagement with memorial sites. Akihiro, a minister in his mid-forties from Nagasaki, reflected on how his role as a priest created an inevitable connection between him and the local Christian cemetery, Sakamoto International cemetery. Due to his role as a priest, Akihiro was triggered to remember the history of Christianity in Nagasaki when visiting Sakamoto and feel personally connected to this history.

A: For the Sakamoto International Cemetery, there are the graves of believers around this church there, and as a priest I go there to lay people’s ashes to rest as a part of my job. It goes without saying that my going to the cemetery is inevitable. There was a priest who used to work in Nagasaki called Mr. Debison who was a part of the Dejima Methodist Church and one of the founders for Shinzai Academy and Kasui Academy. This church used to be a part of the Dejima Methodist Church too, but the previous building burnt down 50 years ago so they used the old wood to create the building you see here. It was Mr. Debison who helped this church be built, and his grave is also in the Sakamoto International Cemetery. In that sense, visiting that cemetery enables us to learn and remember how Christianity came to be in Nagasaki, and to commemorate it. Mr. Debison spent a lot of time all over the country but his wife passed away and was buried in Nagasaki, so after spending some time traveling to Yokohama, America and around the world, he decided to come back to be buried with his wife in Nagasaki in the end. As someone who is a part of this church, I have many personal feelings about that cemetery because it helps to remember that such a person lived and played such a major role in our history.

Identifying as a priest created a special connectedness felt by Akihiro when visiting the cemetery. While all participants in this study identified as ethnically Japanese, only a few simultaneously identified with specific roles and related responsibilities. Participants described how identifying with these roles was their doorway into interacting with certain memorial sites. Tohru, a 40-year-old man residing in Nagasaki, reflected on how his responsibility of recording the testimonies of atomic bomb survivors led to his visiting Sakamoto international cemetery.

T: Sakamoto Cemetery, I went there with one of the Hibakushas (survivors of the atomic bombing). He is a story teller about his experience of atomic bombing and he is well versed in the history of Nagasaki and the history of atomic bombing. He introduced me to go there because we can learn a lot in the cemetery about the history of Nagasaki. A lot of people from foreign countries were there after they passed away, so that’s why I went there with the man.

Akihiro felt that his role as a recorder of the testimonies of hibakusha directly influenced his visiting and understanding of Sakamoto international cemetery. He recognized how much his friend played a role in sharing and in turn, perpetuating his understanding and conceptualization of this memorial site. For Akihiro, his friend’s interpretation of the cemetery influenced his own understanding of it as a site of historical significance in Nagasaki. Incorporating his friend’s narrative of the cemetery was central to Akihiro’s own understanding of its significance for Nagasaki residents.

Yumi, an 80-year-old woman from Hiroshima, similarly shared how her status as a close distance survivor of the atomic bombing impacted her engagement with Hiroshima peace park and commemorative activities taking place on the day of the bombing.

Y: I get a notice from the city every year telling me to come to the memorial service. When I visited with the invitation, they let me sit at the very front at the reception because I’m a close-distance survivor. Since then I’ve always sat at the very front, during the ceremony too. The notice came this year too.

Whether religious, secular, or related to the bombing, stated roles and statuses incited participants to identify with more complex identities reflective of their lived experiences. Their differing experiences and narratives reflect the intractable challenge of managing multiple identities simultaneously when engaging with these memorial sites.

### Sense of duty to pass on accounts of the bombing

As much as customary processes of mourning were enacted to commemorate lives lost during the atomic bombing, participants expressed additional methods of remembering. One such method was sharing and preserving the narratives of atomic bomb victims, primarily so the impact of the bombing would not be forgotten.

Koharu, a 70-year-old woman living in Hiroshima, identified several ways she was engaged in preserving and transmitting the memories of atomic bomb victims. Koharu reflected this commitment by voluntarily recording and sharing the memories recounted by deaf survivors of the atomic bombing.

K: They [deaf atomic bombing survivors] often tell me about their experience of the atomic bomb without being able to hear anything. These almost 90-year-old men and women tell me in great detail using sign language, all about what happened on the 6th of August. I used to live in a different prefecture, so I didn’t think it had much to do with me and I never used to be very interested, but after learning about the experiences of these people, I now strongly believe that it is our duty to pass on these tales. So every year on the 6th of August at 8:15 I always make sure to calm my feelings and put my hands together. In this day and age, many people, including my own daughter, tend to forget the importance of this day, so it is our responsibility to pass on the narratives of the people who experienced the atomic bombing.

This form of memory transmission reflects a shared commitment to conveying the facts of the bombing and increasing awareness and appreciation for the impact of this event. Participants in both cities shared their perceptions of the importance of this form of memory transmission, reflecting feelings of responsibility and duty in passing on accounts of the bombing. Tomohiro, a high school teacher from Nagasaki in his mid-forties, reflected on the importance of sharing these narratives for the purpose of ensuring that they would not be repeated.

T: So many lives were lost on that day of the bombing, and Nagasaki is currently the last place that has been bombed by the atomic bomb, so with that in mind, I think it’s our duty to tell the world that it must never be repeated again.

### Influence of the deceased when making decisions

When discussing deceased family and friends, participants conveyed these memories by reflecting on their experiences interacting with the deceased, particularly regarding the lessons learned from these individuals. Tomohiro reflected on the lessons he learned from a former teacher, discussing how he would often remember him when considering what to do in his own role as a high school teacher.

I: Do, do you feel upset by the memories of the people you have lost?

    T: No, I don’t.

    I: Why is that?

T: I don’t get upset, but I think, "What would my teacher have done in my place?

For Tomohiro, reflecting on what his teacher would have done in his place created opportunities for him to recall and reflect on the role that his friend played in his life. Memories of the former teacher were triggered when Tomohiro would need to make decisions in situations where the deceased would have previously offered advice. In making decisions based on what he thought his teacher would do, Tomohiro transmitted the memory of his teacher by acting in a way that he felt his teacher would have advised and approved of. In this way, Tomohiro’s former teacher was able to continue to influence his current lived experiences.

A similar experience was described by Hinata, an 80-year-old survivor of the atomic bombing from Nagasaki. When describing times when she remembered her deceased friend, Hinata recalled remembering her when making decisions.

I: When you remember those who have passed, what kind of memories in particular do you often remember?

H: When I’m feeling stuck, I remember things like “Oh, if she were here, what kind of advice would she give me” and whatnot. So even when I’m in a debate, I remember things like “if this were her, she would have said something like this.”

### Mourning through celebration

Many participants shared that feelings of sadness or deep loss were not applicable to them or others within their communities. Similarly, regret and negative feelings were shared as not being encouraged at public mourning events, such as Obon. Marika, an 80-year-old woman from Nagasaki, referenced the ways that commemorative events in Nagasaki encourage participants to reflect on those who have passed with enjoyment, celebrating their lives with others.

M: In Nagasaki, there is a famous festival called “Shorongashi’’ to send off those who died the previous year. I don’t know if it’s appropriate to say festival, maybe event. Tourists come from all over the country and it’s very lively. Even though we’re sending off the dead, everyone is having fun. Nagasaki is generally a place that likes to have fun. So even though it’s sad, the children like being able to send everyone off in a fun way during Shoronagashi, and there’s something about being able to watch that.

When reflecting on the deceased for the purpose of remembering, participants like Marika were compelled to share with others in a way that was positive, nostalgic, and joyous. For these reasons, the loss of a loved one did not equate solely with sadness for many participants.

### Engaging in activities that remind of the deceased

The process of sharing about lost loved ones during the interviews prompted participants to reflect on their memories of the deceased’s dispositions and attitudes. Participants from both cities displayed a detailed and holistic memory of their loved one’s personal qualities, identifying links between these qualities and their closeness to them. When reflecting on his late great grandmother, 19-year-old Kyo discussed her personal warmth as a disposition that comforted him.

K: As a family member she was a very warm presence, or, rather than warm… She always listened to us talk, and of course she listened to our stories but she always taught us kids important lessons through her own stories. In that sense I think she was a warm presence.

However, some participants reflected on the qualities of the deceased that made them difficult to be around in life and commemorate in death. Izumi, a 90-year-old survivor of the bombing from Hiroshima recalled the strict nature of her mother who passed away.

I: She was a very righteous person and was strict to our neighbors as well. She wouldn’t hesitate to go into other’s houses and tell them off for things. I used to be embarrassed and would plead with her not to do it, but she would not budge. She would tell me that that was just the way she was, and it was my job to go and apologize to the neighbors for my mother’s actions. I would have to go and apologize so many times.

Izumi described the disposition of her mother as did other participants. However, the focus was placed on the negative impact that her mother’s disposition had on her while she was growing up, which did not translate into a positive feeling associated with the loss. While other participants found it difficult to express sad or angry feelings when recalling the deceased, Izumi and two other participants reflected openly on their feelings of loss and discontent.

Outside of mourning practices taking place at designated dates and times, participants in both Hiroshima and Nagasaki engaged in specific activities they associated with the deceased, believing that engagement with these activities brought them closer to the deceased. For Tomohiro, remembering the life of his deceased former teacher went beyond remembering during Obon or while praying. He described listening to certain music that reminded him of his teacher, including songs they had recorded together when he was alive. These activities were taken a step further when Tomohiro decided to organize a memorial concert in remembrance of his lost teacher.

T: When I listen to certain music, I remember when I played with that teacher. It’s a recording, so when I heard the recording we played before, I remember that time. Last year, I did a memorial concert for the teacher with my friends.

Beyond the rituals typically practiced to commemorate a lost life in Japan, Tomohiro was also compelled to engage in additional activities to pay tribute to the life of his former teacher. Mourning at the designated times and dates did not fully capture the nuances of his grief processes and methods of conveying the memories of his former teacher.

Koharu, a female survivor of the atomic bombing in her mid-eighties, considered preparing the recipes taught to her by a deceased friend. The nature of activities she shared with the deceased compelled Koharu to remember her lost friend in ways outside the bounds of typical methods of grieving, given instead to engage in experiences unique to the relationship she shared with this friend.

K: She used to teach us how to cook too. Now is not the season for Goya, but she would tell us the best ways to prepare this bitter vegetable and she would give us some, too. When this season rolls around, I sometimes think maybe I’ll try to prepare one of her recipes again.

By engaging in individualized activities to remember those lost, participants challenge simplified conceptions of mourning processes. Instead, these participants opted for more complex processes of remembering the deceased which were outside of the typical methods of expressing grief.

## Discussion

These findings indicate several overlapping methods of transmitting memory about death and loss among participants. Overall, the results of these analyses provide evidence that residents of both Hiroshima and Nagasaki engage in generalizable processes of memory transmission about death. Participants valued the opportunity to reflect on the lives of those they lost and often expressed gratitude for their ancestors, including those who have died. Engaging with mourning activities and designated times and places allowed for routine expression of grief for most participants, and there were others who preferred to share the memories of the deceased in an individualized fashion.

Assumptions that Obon and other mourning rituals were routine were shared by several participants who first expressed uncertainty about how to articulate the importance of these activities. This is consistent with previous findings that Japanese mourners do not perform these rites to abide by rules of a larger social structure, but out of a shared feeling of connectedness with the deceased [24, 25]. Participants, hence, described their performance of ancestor rites as a means of feeling connected to ancestors without having given prior thought as to the reasons why. Additionally, in transmitting memories about grief during specific times and dates, participants were able to find a channel for their grief that was readily accessible and widely accepted within their communities.

Participants reported feeling connected and grateful to ancestors, reflecting a central component of ancestor worship, including engagement with rituals which allow for a continued connection to the dead [26]. The Obon holiday is a central component of ancestor worship rituals wherein people believe that spirits of their ancestors come home, reuniting with their family. In Japan, there exists a recognition of the continuity between life and death. Many Japanese residents believe that a person’s spirit belongs to the same family and the same local community before and after death [27]. These perspectives have an important implication on how memory transmission of the dead is enacted in Japan. The continuity that is perceived between the living and dead causes ancestors to be viewed as the primary conduits of transmitting narratives of the deceased, which are fomented in the processes of ancestor worship. Research of grief in Japanese culture suggests that individuals are encouraged to remember the dead fondly, focusing on their good qualities and things they enjoyed about being with them [28, 29]. Even so, some participants felt comfortable sharing their conflicting feelings about those who died, including Akihiro, who reflected not feeling that he has recovered from the suicide of his sister. These results ask us to look past simplified depictions of cultural grief and consider the individual elements that may impact a person’s remembrance, including negative feelings towards the deceased or experiencing a traumatic death.

Memorial sites were viewed as important places not only for the mourning of those lives lost to the atomic bombing, but to mourn loved ones across generations. Many participants in both cities reflected that they visit the memorial sites for the bombings for other activities, such as celebrating Obon, family gatherings, or commemorating the lives of more recently lost loved ones. These findings are consistent with other conceptualizations of memorial sites as focal points for local and national identity, becoming places which hold the memories of the past trauma interwoven with present-day perspectives and uses [30–32].

The sense of duty to pass on the accounts of survivors of the bombing was shared among participants of different age groups and across generations. Preserving the narratives of atomic bomb victims so the impact of the bombing would not be forgotten was viewed by many as crucial to their own grieving processes in relation to the memorial sites. These findings indicate that preserving the memories of the atomic bomb victims was a component of the participant’s own coping and grieving processes. This finding possesses epistemological similarity with the concept of proxy agency, where a representative with power or access to resources can act on a person’s behalf to achieve outcomes [33, 34]. By taking agency following a traumatic event, individuals may begin to feel a sense of control over the events which caused the trauma. This can contribute positively to the grieving process both on the individual and collective levels.

Memory transmission of the deceased was viewed by several participants as not saddening, but celebratory and joyous. Participants reflected feeling a happy form of nostalgia when reflecting on their lost loved ones, appreciating the opportunities they had to mourn and remember them. Participants also expressed dismay when asked if remembering their loved ones brought on any negative feelings, such as sadness or depression. This finding is consistent with findings from previous research, which refers to this form of memory transmission in Japanese society as ‘cherished reminiscence’ of the deceased [21, 22]. Cherished reminiscence has previously been investigated as an aspect of the mourning process which may facilitate adjustment to loss [35, 36]. The interviews from this study support these findings, as participants reflect that their positive remembrance of the deceased leaves them feeling connected, hopeful, and at peace.

An unexpected finding of this study was the interview itself serving as an intervention that encouraged the transmission of memory about the deceased. By asking participants to articulate their reasons for engaging in certain mourning activities, they were encouraged to articulate on the importance of said activities and convey this to the interviewers. Additionally, by asking questions about their feelings about those they’ve known who have died, participants were prompted to recall the memory of these individuals and transmit it orally. A particularly potent example of this was the memories transmitted by hibakusha regarding their experiences of surviving the atomic bombing. While participants were not prompted to recount these experiences, almost all who were hibakusha chose to re-tell these experiences in extreme detail. Marika, for example, chose to describe the impact that the bombing had on her father, who was a direct survivor of the bombing. Bringing books and photos, she shared with the research team her family’s experiences.

M: My father is still active as a storyteller himself, and he can talk about it easily because it’s his own experience. However, since I haven’t experienced the war or the bombing, it’s very difficult for me to tell this story. I go around using this picture-book play that was made based on a memoir by my father.

M: (showing the researchers photos of her family before the bombing in the picture book). Looks similar? This is the bombing site, and here is my father’s house. We’re here now. It was only 800 meters away. Everything turned into ash. Here are the siblings—father, mother, sister, my father, younger sister. Brother, sister, brother. All of them died. All of these siblings died in the atomic bombing. My father cremated his 3-year-old brother by himself.

Participants used the interview space to share the memories of the deceased that they felt were important to convey to the research team. In this way, the research itself continued the process of memory transmission by extending the sharing of these memories to readers and others in the field.

## Limitations and directions for future research

Placing the transmission of memories about death among individuals at the center of analysis, this study investigated the methods of remembering expressed by participants in Hiroshima and Nagasaki. Yamamoto described how the deceased remains accessible to Japanese mourners and speaking and offering tributes to ancestors is common [19]. In addition, the identification of one’s grieving processes and thoughts about death is an important part of forming and then transmitting memories of the deceased. Participants of this study, as individuals of differing ages, genders, and cities of residents, nonetheless shared methods of transmitting memory about the deceased that were found to be common among them. Able to be easily located within memory transmission, certain activities and narratives developed by participants established a common discourse of remembering that combined traditional methods of remembering to those that were specific to the relationship between the living and the deceased. In developing their own methods for remembering, participants emphasized the aspects of the deceased that were important to them, including their personal qualities, their status as survivors of the atomic bombing, or activities they once shared together.

There are two limitations to this study. The first regards the small sample size for qualitative data analysis. A small sample was selected to allow for in-depth analysis and engagement, along with accounting for the complexity of interpreting and translating the interviews from Japanese to English. However, the smaller sample size did not impact the ability to perform the qualitative data analysis and glean themes from the interviews. Future studies using this design could employ a larger sample to validate results.

The second limitation regards the reliance of interviews from a single time point rather than longitudinal analysis, which would involve interviewing individuals at different points in time. However, given that this is the first known qualitative study regarding the mourning and memory transmission experiences of individuals in an area impacted by mass trauma, this research presents an important contribution to knowledge of memory transmission in the process of grieving. Future studies in this area could consider researching the prevalence and persistence of bereavement practices among individuals residing in an area impacted by mass trauma through the collection of longitudinal data collected at distinct time points.

The results of this study have important implications for the field of Japanese studies, guiding academics in ways to articulate the methods of sharing and transmitting memories among Japanese citizens in these two cities. Particularly when working with persons who have experienced loss due to violence or politics, previous research has deemed it critical to avoid pressuring individuals to engage in memory making activities that are inappropriate for them on personal, cultural, or spiritual grounds [37–39]. The findings of this study have a culturally specific grounding for individuals residing in Japan, along with the specific experiences of individuals who reside in a location impacted by mass trauma. Therefore, while these results may not be generalizable across demographics, they provide a way forward in researching the memory transmission and mourning experiences of individuals impacted by mass trauma. They also provide a space to discuss these experiences as they relate specifically to the experience of individuals residing in Japan. Future research in Japanese studies can replicate these results with other individuals in these cities, or in other locations in Japan that have experienced differing forms of mass trauma, such as Fukushima, which was impacted by a nuclear accident in 2011.

This study also has important implications for individuals working in the grief and bereavement fields as either researchers or social service providers. For researchers, the results of this study indicate distinct methods of processing grief and remembering the deceased for individuals residing in areas impacted by mass trauma. These findings may be useful in the research of other similar populations, such as those living in areas impacted by mass violence, war, or terrorism. For social service providers, the findings of this study indicate pathways for grieving that are culturally responsive for Japanese individuals which may help avoid prolonged or complicated grief. Social service providers and therapists working with individuals from such communities may benefit from understanding how the memory transmission around grief may take place for such individuals and incorporate these methods into their treatment plans.

Investigated in this study was the role that societal mechanisms and institutions play in the transmission of memory between individuals to groups and the larger community. All participants reflected on the importance of the presence of certain memorial sites in being able to share their grief with others and the deceased. This is particularly important when considering the silencing of grief memories that take place following mass violence, particularly those events occurring from war or political events [40–42]. Future studies could focus on the specific role that differing types of memorial sites and other mechanisms have on the transmission of memories related to death.

## Conclusion

The forms of memory transmission identified among residents of a community impacted by mass trauma and violence provides new perspectives on the ways that mourning is experienced by individuals. Old assumptions that Japanese people experience and transmit feelings of grief through the same traditions were disrupted in this study. The results of this study will hopefully lead to critical engagement with the mechanisms of memory transmission that participants communicated in their narratives. These experiences included sharing with others about the characteristics of the deceased, celebrating, interacting with memorial sites, and engaging in activities that bring up memories of the deceased. The analyses presented here will hopefully serve as a promising beginning to future research into the methods of memory transmission among individuals within communities impacted by intergenerational trauma caused by mass violence. By supporting the development and continuation of such research of individual-level capacities and skills in transmitting in sharing memory, this research might contribute towards efforts of understanding the formation of collective memories.

## Supporting information

S1 Text Inclusivity in global research. (DOCX)
